# Insertable cardiac monitor with a long sensing vector: Impact of obesity on sensing quality and safety

**DOI:** 10.3389/fcvm.2023.1148052

**Published:** 2023-03-21

**Authors:** Giovanni Bisignani, Silvana De Bonis, Bertrand Pierre, Dennis H. Lau, Daniel Hofer, Victor Manuel Sanfins, Andreas Hain, Pilar Cabanas, Eimo Martens, Antonio Berruezo, Romain Eschalier, Paul Milliez, Ulrich Lüsebrink, Jacques Mansourati, Georgios Papaioannou, Daniele Giacopelli, Alessio Gargaro, Sylvain Ploux

**Affiliations:** ^1^ Department of Cardiology, Ospedale Civile Ferrari, Castrovillari, Italy; ^2^ Department of Cardiology, CHRU de Tours, Tours, France; ^3^ Department of Cardiology, Royal Adelaide Hospital, Adelaide, SA, Australia; ^4^ Department of Cardiology, Universitätsspital Zürich, Zurich, Switzerland; ^5^ Department of Cardiology, Hospital Senhora da Oliveira—Guimarães, Guimarães, Portugal; ^6^ Department of Cardiology, Kerckhoff-Klinik GmbH, Bad Nauheim, Germany; ^7^ Department of Cardiology, Hospital Álvaro Cunqueiro, Vigo, Spain; ^8^ Department of Cardiology, Klinikum Rechts der Isar der Technischen Universität München, München, Germany; ^9^ Department of Cardiology, Centro Médico Teknon, Barcelona, Spain; ^10^ Department of Cardiology, Hôpital Gabriel Montpied, Clermont Ferrand, France; ^11^ Department of Cardiology, Le Centre Hospitalier Universitaire de Caen CHRU Caen, Caen, France; ^12^ Department of Cardiology, Universitätsklinikum Gießen und Marburg GmbH, Standort Marburg, Germany; ^13^ Department of Cardiology, CHU de Brest, Brest, France; ^14^ Department of Cardiology, Hôpital Saint-André, Bordeaux, France; ^15^ Clinical Unit, Biotronik Italia, Milano, Italy; ^16^ Department of Cardiac, Thoracic, Vascular Sciences & Public Health, University of Padova, Padova, Italy; ^17^ Hôpital Haut Lévêque (CHU), Bordeaux, France

**Keywords:** insertable cardiac monitor, implantable loop recorder, obesity, r-wave amplitude, long-sensing vector, signal quality

## Abstract

**Background:**

Fat layers in obese patients can impair R-wave detection and diagnostic performance of a subcutaneous insertable cardiac monitor (ICM). We compared safety and ICM sensing quality between obese patients [body mass index (BMI) ≥ 30 kg/m^2^] and normal-weight controls (BMI <30 kg/m^2^) in terms of R-wave amplitude and time in noise mode (noise burden) detected by a long-sensing-vector ICM.

**Materials and methods:**

Patients from two multicentre, non-randomized clinical registries are included in the present analysis on January 31, 2022 (data freeze), if the follow-up period was at least 90 days after ICM insertion, including daily remote monitoring. The R-wave amplitudes and daily noise burden averaged intraindividually for days 61–90 and days 1–90, respectively, were compared between obese patients (*n* = 104) and unmatched (*n* = 268) and a nearest-neighbour propensity score (PS) matched (*n* = 69) normal-weight controls.

**Results:**

The average R-wave amplitude was significantly lower in obese (median 0.46 mV) than in normal-weight unmatched (0.70 mV, *P* < 0.0001) or PS-matched (0.60 mV, *P* = 0.003) patients. The median noise burden was 1.0% in obese patients, which was not significantly higher than in unmatched (0.7%; *P* = 0.056) or PS-matched (0.8%; *P* = 0.133) controls. The rate of adverse device effects during the first 90 days did not differ significantly between groups.

**Conclusion:**

Although increased BMI was associated with reduced signal amplitude, also in obese patients the median R-wave amplitude was >0.3 mV, a value which is generally accepted as the minimum level for adequate R-wave detection. The noise burden and adverse event rates did not differ significantly between obese and normal-weight patients.

**Registration**: URL: https://www.clinicaltrials.gov; Unique identifier: NCT04075084 and NCT04198220.

## Introduction

Insertable cardiac monitors (ICMs) are increasingly used for the diagnosis of heart rhythm disorders (1). ICMs allow efficient investigation of palpitations, unexplained recurrent syncope or cryptogenic stroke, assessing arrhythmia relation (2). Moreover, sophisticated ICM algorithms capable of detecting atrial fibrillation (AF) episodes are used to guide clinical management of AF (3, 4). ICM electrodes are placed under the skin to record a subcutaneous electrogram (sECG). The absence of direct electrode contact with the heart muscle and factors such as patient weight and implant location can reduce the R-wave amplitude, cause interference, and generate electrical noise, impairing R-wave detection and diagnostic performance of the ICM (5). Fat layers can further reduce detection accuracy by increasing the distance of the electrodes from the heart. This is even more important as obesity was found to be an independent clinical predictor of pacemaker implantation and atrial arrhythmia in patients with ICM (6, 7). Recently, devices with long sensing vectors have been designed to obtain larger *P*- and R-wave amplitudes and thus improve sECG quality compared to standard sensing vectors (8). However, there are still no data on the ICM sensing performance and the safety of ICM insertion in obese vs. non-obese patients.

## Materials and methods

### Study objective and patient selection

The purpose of the present analysis was to compare sensing performance and safety of a long-sensing-vector ICM between obese and non-obese patients using data from the ongoing BIO|STREAM.ICM registry (ClinicalTrials.gov identifier: NCT04075084) and the completed BIO|STREAM.ICM “Obesity” submodule (NCT04198220). These multicentre, prospective, non-randomized studies were initiated to collect data during routine care of patients with a standard indication for ICM implantation. Investigational sites accepted a central Ethics Committee's vote for the respective study or obtained a separate local approval according to national regulations. All patients gave their written informed consent for study participation.

Patients were included in the present analysis if their body mass index (BMI) was known and the follow-up period after device insertion was at least 90 days, including remote monitoring. Available data by January 31st, 2022 (time of data freeze) were considered for the analysis.

### Device

As previously described (9, 10), the BIOMONITOR III (BIOTRONIK, Berlin, Germany) is an ICM characterized by a rigid housing and a flexible antenna with an electrode on its tip. This specific design extends the sensing vector from ≈45 mm to ≈70 mm, to enhance signal amplitude while maintaining a cross-sectional profile and ICM weight similar to that of ICMs without antenna (1). A dedicated insertion tool allows for a single-step injection-like implantation of BIOMONITOR III into the subcutaneous tissue.

The device continuously evaluates cardiac rhythm based on R-R intervals. Depending on programmed parameters, up to five different types of arrhythmias can be automatically detected and documented with high-resolution sECG recordings, additional episode-related data, and long-term diagnostic data. The noise burden is automatically quantified by the device as the proportion of a 24-hour period, in which the device detects interfering noise signals due to high frequency signals (<180 ms). Noise signals inhibit automatic arrhythmia detection. The data are transmitted by the BIOTRONIK Home Monitoring® system on a daily basis without patient involvement and can be accessed by the responsible physician at any time using a secure internet platform.

### Endpoints and data analysis

The study cohort was divided into an “obese” group (BMI ≥30 kg/m^2^) and a normal-weight control group (BMI <30 kg/m^2^). The quality of the sECG was compared between the groups in terms of R-wave amplitude and amount of noise and artifacts. The mean R-wave amplitude is automatically determined by the device each day. Remotely transmitted values in individual patients were averaged for the period from day 61 to day 90 after device insertion and denoted as the “3-month average R-wave amplitude”. Daily noise burden is defined as the percentage of a day during which high-frequency signals prevent rhythm analysis. It was averaged per patient for the period of 90 days from insertion. The number of patients with a noise burden >5% on at least one day during this period was counted. Also, adverse device effects, defined as any untoward medical occurrence related to the use of the ICM, were assessed and compared between groups.

### Statistics

Descriptive statistics were reported as median with interquartile range for continuous variables and as absolute and relative frequencies for categorial variables. Differences between groups were tested with the Mann-Whitney U-test (continuous variables), the Fisher's exact test (binary variables), and the Chi-squared test (categorical variables with >2 levels). To minimize confounding effects in the non-randomized comparison, we performed a 1:1 nearest-neighbour propensity score (PS) matching by selecting a subset of matched normal-weight controls (the “PS-matched control group”) based on the average treatment effect on treated (obese group). As expected, a number of prespecified clinical covariates with known correlation with increased BMI (history of AF, hypertension, sleep apnoea, diabetes mellitus) were imbalanced between the obese and normal-weight groups and were selected as matching covariates along with implant angle (parasternal vs. along heart axis), age, and sex. After PS-matching, the residual difference was tested for all baseline characteristics. Uni- and multivariable linear regression models adjusted for known prognostic factors were used to model the association between the inverse BMI and the amplitude of the R-wave, to emphasize the inverse relation. We also generated logistic models to estimate the odds ratio and the 95% confidence interval (CI) for the incremental risk of a noise burden >5% on at least one day during 90 days of follow-up in obese vs. control patients. Statistical significance was defined as *P* < 0.05. All statistical analyses were performed using the STATA statistical software package (version 17.0, StataCorp LP, College Station, TX, USA).

## Results

### Study population

The present analysis included 372 patients from 29 sites in 9 countries (Supplementary Table S1). The obese and control groups accounted for 104 (28.0%) and 268 (72.0%) subjects, respectively. Compared to controls, obese patients were older (68.5 [57.0–76.5] vs. 64.0 (51.0–74.0) years, *P* = 0.027), had cryptogenic stroke less frequently as ICM indication (14.4% vs. 28.4%, *P* = 0.024), and had more comorbidities (hypertension, diabetes, valvular disease, history of AF, sleep apnoea) (Table 1). Implant angle (parasternal or along heart axis) did not differ significantly between groups (*P* = 0.682).

**Table 1:** Patient characteristics at the time of ICM insertion.

Variable	Obese group (A)	Control group (B)	PS-matched control group (C)	*P*-value^a^	*P*-value^a^
	*N* = 104	*N* = 268	*N* = 69	A vs. B	A vs. C
Age (years)	68.5 (57.0–76.5)	64.0 (51.0–74.0)	69.0 (55.0–76.0)	0.017	0.747
Female	53 (51.0%)	111 (41.4%)	35 (50.7%)	0.104	1.00
BMI (kg/m2)	33.0 (31.7–36.0)	25.3 (23.1–27.5)	25.8 (23.7–27.6)	<0.0001	<0.0001
Height (cm]	166 (160–175)	170 (163–178)	170 (162–176)	0.006	0.125
Weight (kg)	96.0 (87.5–105.0)	74 (64–81)	74 (64–85)	<0.0001	<0.0001
**Comorbidities/arrhythmias**
Hypertension	81 (77.9%)	125 (46.6%)	52 (75.4%)	<0.001	0.716
History of cerebrovascular disease	21 (20.2%)	81 (30.2%)	24 (34.8%)	0.053	0.035
Diabetes mellitus	24 (23.1%)	30 (11.2%)	13 (18.8%)	0.005	0.573
History of AF	21 (20.2%)	29 (10.8%)	9 (13.0%)	0.027	0.305
Valvular disease	15 (14.2%)	17 (6.3%)	5 (7.2%)	0.022	0.220
Peripheral vascular/artery disease	5 (4.8%)	18 (6.7%)	5 (7.2%)	0.634	0.522
Chronic renal failure	11 (10.6%)	18 (6.7%)	9 (13.0%)	0.280	0.634
Sleep apnoea	17 (16.3%)	13 (4.8%)	7 (10.1%)	0.001	0.272
History of heart failure	18 (17.3%)	27 (10.1%)	8 (11.6%)	0.075	0.387
CAD	15 (14.4%)	37 (13.8%)	13 (18.8%)	0.869	0.528
Prior MI	4 (3.8%)	17 (6.3%)	7 (10.1%)	0.437	0.411
Chronic obstructive pulmonary disease	7 (6.7%)	11 (4.1%)	4 (5.8%)	0.290	1.00
**ICM indication**				0.024	0.127
Syncope/presyncope	66 (63.5%)	156 (58.2%)	38 (55.1%)		
Cryptogenic stroke	15 (14.4%)	76 (28.4%)	21 (30.4%)		
AF monitoring	9 (8.6%)	10 (3.7%)	3 (4.3%)		
Palpitation	5 (4.8%)	7 (2.6%)	2 (2.9%)		
Other	9 (8.6%)	19 (7.1%)	5 (7.2%)		
**Implant angle**				0.682	0.921
Parasternal	40 (38.5%)	88 (32.8%)	23 (33.3%)		
Along heart axis	64 (61.5%)	179 (66.8%)	46 (66.7%)		

AF, atrial fibrillation; BMI, body mass index; CAD, coronary artery disease; ICM, implantable cardiac monitor; MI, myocardial infarction; PS, propensity score.

^a^ Results of Mann-Whitney *U*-test (continuous variables), Fischer's exact test (binary variables), and Chi-squared test (categorical variables with >2 levels).

The PS-matching identified a subset of 69 matched normal-weight controls and reduced the mean bias for the selected covariates by 73.6% (Supplementary Table S2 and Supplementary Figure S1). After PS-matching, no patient characteristic at baseline, except for BMI and weight, differed significantly between the obese group and matched controls (Table 1).

### R-wave amplitude

**Figure 1:**
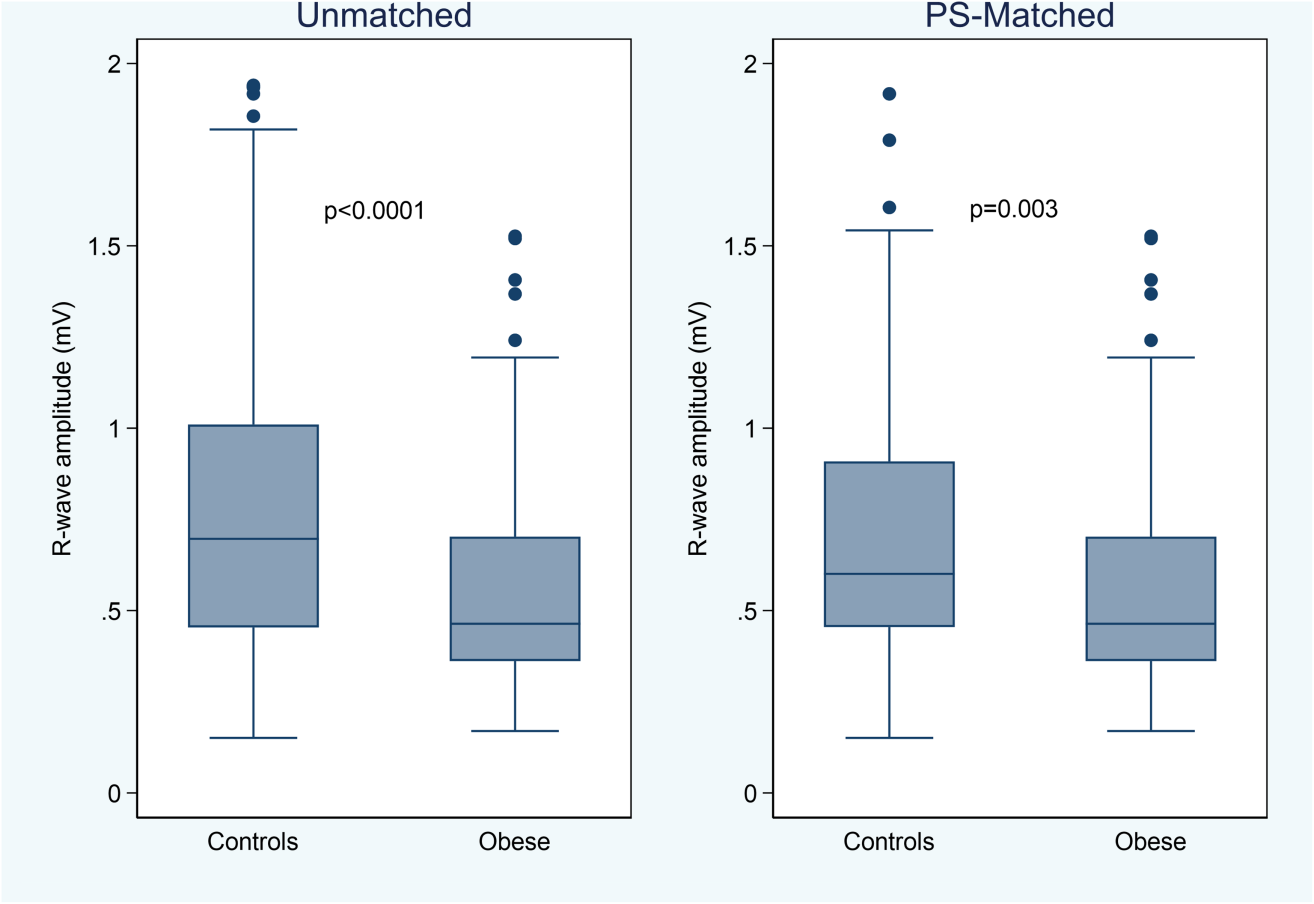
Box-whiskers plot of the 3-month average R-wave amplitude by study groups, in the unmatched and propensity score (PS) matched cohorts.

The 3-month average R-wave amplitude was significantly lower in the obese group [0.46 (0.36–0.70) mV] than in the control group [0.70 (0.45–1.01) mV, *P* < 0.0001] or in the PS-matched control group [0.60 (0.45–0.91) mV, *P* = 0.003] (Figure 1). However, also in obese patients, the median R-wave amplitude was significantly higher than 0.3 mV (*P* < 0.001), a value which is generally accepted as the minimum level for adequate R-wave detection (11), and 18 (17%) patients had a lower value as compared to 6 (9%) in the PS-matched control group (*P* = 0.122).

**Figure 2:**
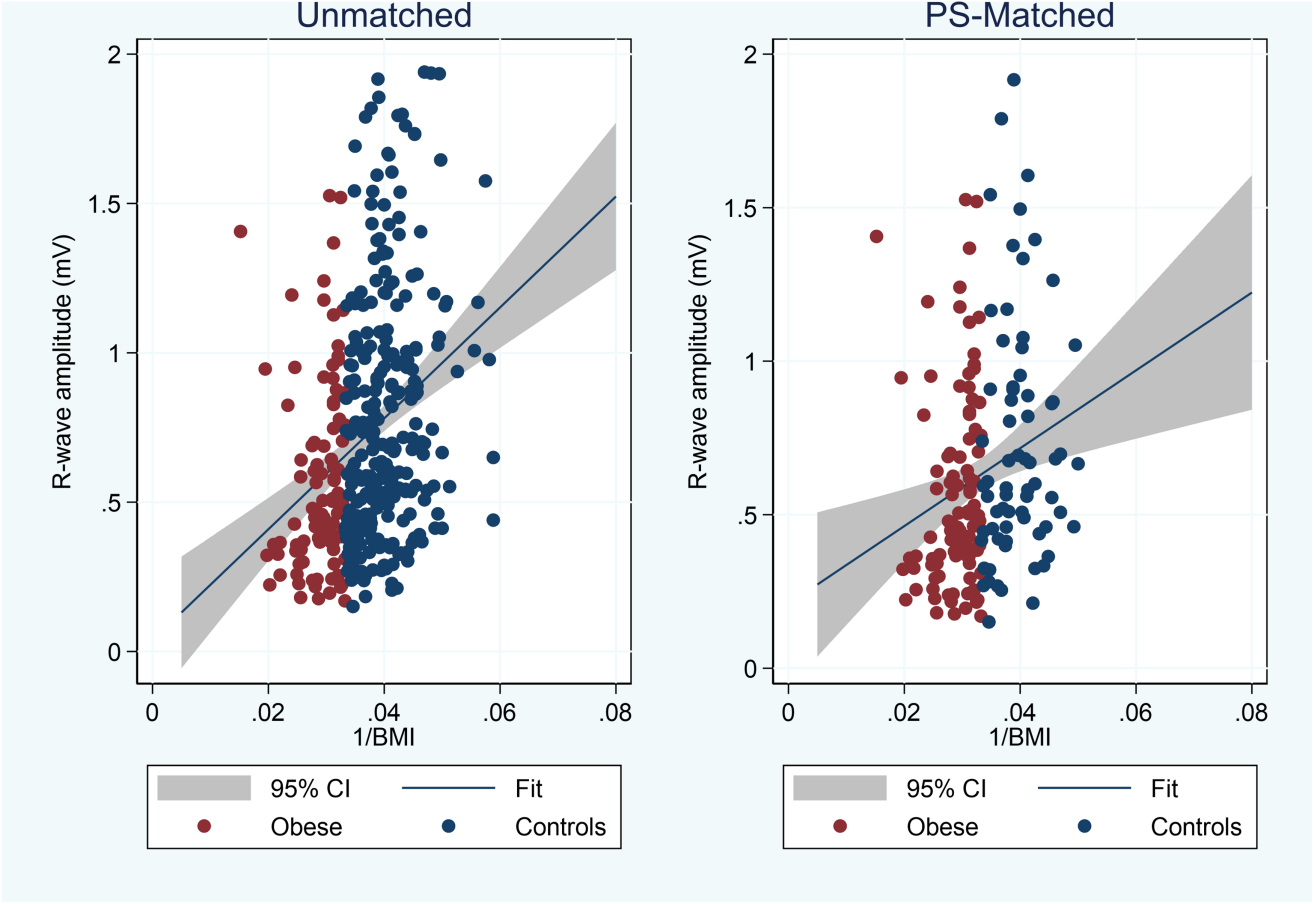
Plot of average 3-month R-wave amplitudes vs. inverse BMI values and linear fit (blue line) with 95% confidence interval (CI) in unmatched and propensity score (PS) matched cohorts.

In Figure 2, we plotted the average R-wave amplitudes against the inverse BMI values (=1/BMI). BMI was significantly correlated with reduced R-wave amplitude also after adjusting for age, sex, and implant angle (Table 2). Interestingly, younger age and implant along the heart axis (as compared to parasternal position) remained associated with larger R-wave amplitudes also after PS-matching, in contrast to sex (Table 2).

**Table 2:** Linear regression analysis of 3-month average R-wave amplitude vs. BMI.

Unmatched cohort^a^				
*N* = 372	Univariable		Multivariable	
	F = 41.4 *P* < 0.0001 R-squared = 0.10		F = 24.7 *P* < 0.0001 R-squared = 0.25	
	Regression Coefficient (95% CI)	*P*-value	Regression coefficient (95% CI)	*P*-value
1/BMI	18.6 (12.9–24.2)	<0.001	15.7 (10.3–21.1)	<0.001
Age/10	* *	* *	−0.07 (−0.9 to −0.05)	<0.001
Female	* *	* *	−0.17 (−0.26 to −0.08)	<0.001
Implant angle (parasternal vs. along heart axis)	* *	−0.25 (−0.35 to −0.15)	<0.001
PS-matched cohort (*N* = 173)^b^
	F = 9.6 *P* = 0.0023 R-squared = 0.05		F = 6.3 *P* < 0.0001 R-squared = 0.16	
	Regression Coefficient (95% CI)	*P*-value	Regression coefficient (95% CI)	*P*-value
1/BMI	12.7 (4.6–20.8)	0.002	11.9 (4.1–19.6)	0.003
Age/10	* *	* *	−0.04 (−0.08 to −0.01)	0.015
Female	* *	* *	0.01 (−0.12 to 0.13)	0.888
Implant angle (parasternal vs. along heart axis)	* *	−0.17 (−0.32 to −0.02)	0.027

1/BMI, the inverse of body mass index; Age/10, decades of age; CI, confidence interval; PS, propensity score.

^a^ Pooled obese group (*n* = 104) and unmatched control group (*n* = 268).

^b^ Pooled obese group (*n* = 104) and PS-matched control group (*n* = 69).

### Noise burden

**Figure 3:**
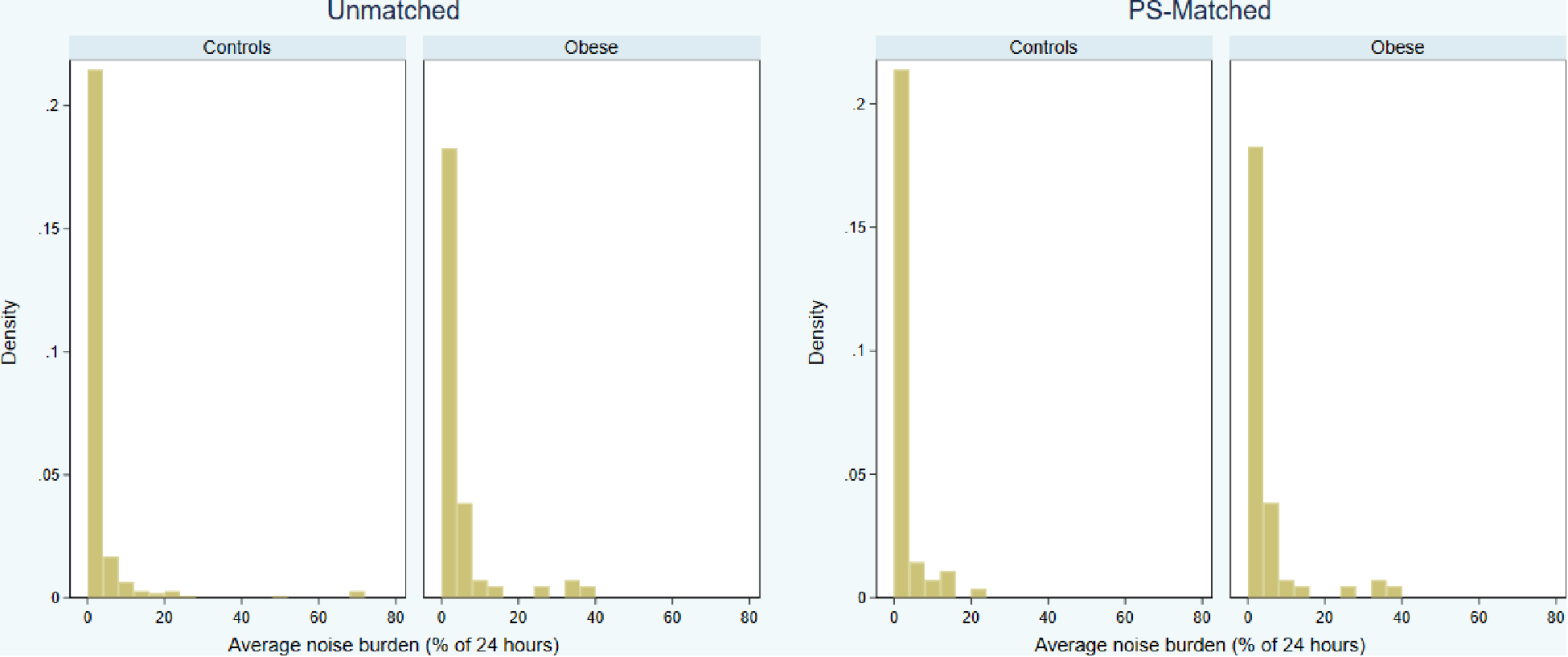
Distributions of 3-month average daily noise burden (percentage of 24 h) by study groups, in the unmatched and propensity score (PS) matched cohorts.

The distributions of daily noise percentage were skewed regardless of study group (Figure 3). In the obese group, the noise burden was 1.0% (0.1%–4.4%), with a trend towards higher noise prevalence compared to unmatched controls (0.7%; 0–2.5%; *P* = 0.056) but not compared to PS-matched controls (0.8%; 0–4.4%; *P* = 0.133). Obesity was not a significant predictor of a high (>5%) noise burden [39/104 vs. 19/69 patients; odds ratio, 1.58 (CI, 0.81–3.06), *P* = 0.175] (Table 3).

**Table 3:** Number of patients with >5% noise burden on any day within 90 days from insertion and the results of the logistic regression analysis.

	Obese group (A)	Control group (B)	PS-matched control group (C)	Odds ratio (95% CI)	*P*-value	Odds ratio (95% CI)	*P*-value
	*N* = 104	*N* = 268	*N* = 69	A vs. B		A vs. C	
Noise >5%, *n* (%)	39 (37.5%)	89 (33.2%)	19 (27.5%)	1.21 (0.75–1.93)	0.435	1.58 (0.81–3.06)	0.175

CI: confidence interval; PS: propensity score.

### Adverse device effects

Nine adverse device effects were reported within 3 months after ICM insertion, none of which was serious. Table 4 shows the type of events by study group. There was no significant difference in the incidence of events between obese patients and controls (*P* = 0.36).

**Table 4:** Adverse device effects during 3 months after ICM insertion (no patient had more than one event).

Adverse effect	Obese group (A)	Control group (B)	PS-matched control group (C)	*P*-value
	*N* = 104	*N* = 268	*N* = 69	A vs. C
Incision site minor bleeding/hemorrhage/haematoma	1	2	2	* *
Implant site pain	1	0	0	* *
Pocket erosion	0	1	1	* *
Infection	0	1	0	* *
Wound healing disorder	0	1	0	* *
Device extrusion	0	1	0	* *
Migration	0	1	0	* *
**TOTAL**	**2 (1.9%)**	**7 (2.6%)**	**3 (4.3%)**	**0.36**

ICM, insertable cardiac monitor; PS, propensity score.

## Discussion

### Main findings

We investigated the impact of increased BMI on sECG quality and the rate of device-related complications in patients undergoing ICM insertion. Obese patients exhibited a significantly lower R-wave amplitude than normal-weight controls even after balancing study groups by prespecified demographics and clinical covariates using a PS-matching method. However, R-waves were significantly larger than 0.3 mV (the minimum targeted sensing value^11^), while the noise burden on the sECG did not increase significantly with BMI as compared to PS-matched controls.

### Sensing pitfalls in ICM

There is a greater tolerance for artifacts and sensing quality in ICMs compared to pacemakers or implantable cardioverter-defibrillators. However, the widespread use of remote monitoring in ICM patients has recently created awareness of the significant impact of false positive alerts for arrhythmias on daily clinical workload (12). Undersensing due to reduced R-wave amplitude is the primary mechanism for false diagnoses of bradycardia or sinus arrest and for false AF alerts (5). sECG has an intrinsic beat-to-beat amplitude variability due to the distance of ICM electrodes from the cardiac muscle, while significant signal drops can be related to body movements and posture changes. Although improvements in automatic algorithms have reduced inappropriate identification of arrhythmias at the cost of slightly reduced sensitivity (13), recent data suggest that false arrhythmia alerts still occur in 20% of implanted ICMs due to inadequate R-wave sensing (5). A good strategy to reduce the burden of false positive alerts is to try to achieve the largest possible R-wave amplitude at insertion, which could potentially decrease the likelihood of undersensing events (12). The inverse relationship of large R-wave amplitudes with false arrhythmia detections was first suggested in a study that reported that patients with larger sECG amplitudes were also significantly less likely to have a high noise burden (14). To obtain large R-wave amplitudes, the ICM model investigated in the present study was designed with an extended sensing vector using a flexible antenna that increases the inter-electrode spacing up to approximately 70 mm, very close to the optimal distance suggested by experimental research (15). A recent study directly comparing long- and standard- (<50 mm) sensing-vector ICMs confirmed that the long vector can be a good solution to obtain large sECG amplitudes (8).

### ICM in obese patients

Obesity is a rising condition with a known association with cardiac arrhythmias, increased risk of sudden cardiac death, and AF (7, 16). Therefore, reliable sensing performance of ICMs also in obese patients is becoming increasingly important. A concern in these patients is that fat layers may either reduce signal amplitudes or increase noise burden and false arrhythmia detection. Therefore, a long-sensing-vector solution with larger R-wave amplitudes and a noise level essentially unrelated to BMI may be the optimal option in obese patients. Although we observed an expected inverse relationship between R-wave amplitude and BMI, the 0.46 mV median amplitude obtained in the obese group was still significantly higher than the 0.3 mV minimum required level with the device we used.

### Comparison with previous studies

The impact of body composition on sensing amplitude detected by the ICM was among the secondary endpoints of several previous studies. Although data from short-sensing-vector devices (Reveal LINQ) suggested an inverse relationship between R-wave amplitude and BMI (17), studies with long-sensing-vector ICMs were less conclusive (8, 10, 18–22) (Table 5). Lacour et al. (18) did not observe such relationship in a small cohort of 19 patients implanted with a previous device version (BioMonitor 2). In a larger population with the same device (*n* = 84), Forleo et al. (19) showed a tendency to lower values in obese people (*P* = 0.074). More recently, Pitman et al. (8) found a significant but weaker negative correlation between BMI and R-wave amplitudes in BIOMONITOR III as compared to standard-sensing-vector devices. While using a PS-matching method to reduce uncontrolled confounders, we confirmed a negative but not critical impact of BMI on R-wave amplitude detected by the long-sensing-vector ICM and relatively unaffected noise levels by BMI.

**Table 5:** Studies on long-sensing-vector ICMs reporting BMI and R-wave amplitude data.

Study	ICM model	No. of patients	BMI (kg/m^2^)*	R-wave amplitude (mV)*	BMI effect
Lacour et al., 2017 (18)	BioMonitor 2	19	27.1 ± 6.7	N.R.	BMI had no statistically significant effect on the amplitude
Bisignani et al., 2018 (prepectoral group) (20)	BioMonitor 2	30	25.4 ± 2.6	0.87 ± 0.44	N.R.
Reinsch et al., 2018 (21)	BioMonitor 2	30	28.9 ± 6.31	1.02 ± 0.47	N.R.
Awad et al., 2020 (22)	BioMonitor 2	77	31.4 ± 6.6	0.77 ± 0.5	N.R.
Forleo et al., 2021 (19)	BioMonitor 2	84	24.6 (22.3–29.0)	1.10 (0.72–1.48)	Obese patients tended to have lower amplitudes compared to normal or underweight subjects (*P* = 0.074)
Deneke et al., 2022 (10)	BIOMONITOR III	653	27.8 ± 5.6 27.0 (24.0–30.6)	0.73 ± 0.40 0.60 (0.42–0.97)	N.R.
Pitman et al., 2022 (8)	BIOMONITOR III	40	27.0 (25.0–28.0)	0.78 (0.52–1.10)	Amplitude was lower with higher BMI. The correlation was weaker with long-sensing-vector ICM.
Present study (obese group)	BIOMONITOR III	104	33.0 (31.7–36.0)	0.46 (0.36–0.70)	Increased BMI was significantly correlated with reduced amplitude.
Present study (control group)	BIOMONITOR III	268	25.8 (23.7–27.6)	0.70 (0.45–1.01)	*** ***

BMI, body mass index; ICM, insertable cardiac monitor; N.R., not reported.

* Data are shown as mean ± standard deviation or median (interquartile range).

### Safety and study limitations

Our study also demonstrated the safety of the device insertion in obese patients in a real-life clinical setting, with a low rate of device-related complications (1.9%), comparable to that in general population (23). As also partially suggested by a recent study (24), patients with high BMI may even be less prone to pocket erosion and spontaneous extrusion of the device, while the migration of the device from the original position may occur more frequently in the adipose tissue. Noteworthy, we did not observe these events, although the short duration of follow-up limits our conclusions on this point. The definition of obesity was based on the BMI which is a well-established method to quantify fat accumulation. However, it does not provide information on sex-specific fat repartition (i.e., android-vs. gynecoid-type obesity) which we tried to mitigate by including sex both in the PS-matching procedure and in the multivariable regression analysis. A further limitation of our analysis is the lack of assessment of P-wave visibility, which is increasingly used among sECG quality testing points, although detection algorithms are still based on R-R interval analysis in current devices. We did not report on comparison of true/false alerts of automatic arrhythmia detection. This would require analysis of performance of each detection algorithm specifically designed for a target arrhythmia, which was beyond our scope. However, we showed that despite inverse correlation of R-wave amplitude and BMI, satisfying R-wave sensing performance (on which arrhythmia detection algorithms rely on) was obtained also in obese patients. Strengths of our study may include the relatively large sample size and the use of PS-matching to mitigate cohort heterogeneity and confounding bias.

## Conclusion

Our PS-matched analysis on a pooled dataset from multicentre prospective studies showed that the use of a long-sensing-vector ICM in obese patients is safe and effective in providing reliable sensing performance. Although increased BMI was associated with reduced signal amplitude, R-wave amplitudes in obese patients were still significantly larger than the 0.3 mV minimum accepted level, and the noise burden was not significantly different compared to normal-weight controls. The incidence of adverse device effects was also similar between groups. Further clinical research is needed to assess the potential impact of increased BMI on the arrhythmia diagnosis performance of ICMs.

## Data Availability

The raw data supporting the conclusions of this article will be made available by the authors, without undue reservation.
