# Supplementary material for: Insertable cardiac monitor with a long sensing vector: Impact of obesity on sensing quality and safety
Source: Front Cardiovasc Med. 2023 Mar 21;10:1148052. doi: 10.3389/fcvm.2023.1148052 (PMC10071510; doi:10.3389/fcvm.2023.1148052)
Supplement: Supplementary file 1 [file Table1.docx]

Supplementary Material

Insertable cardiac monitor with a long sensing vector:

impact of obesity on sensing quality and safety

Giovanni Bisignani^*^, Silvana De Bonis, Bertrand Pierre, Dennis H Lau, Daniel Hofer, Victor Manuel Sanfins, Andreas Hain, Pilar Cabanas, Eimo Martens, Antonio Berruezo, Romain Eschalier, Paul Milliez, Ulrich Lüsebrink, Jacques Mansourati, Georgios Papaioannou, Daniele Giacopelli, Alessio Gargaro, Sylvain Ploux

*** Correspondence:** Giovanni Bisignani: [giovanni.bisignani@virgilio.it](mailto:giovanni.bisignani@virgilio.it)

Contents

[Table S1. List of sites and responsible physicians 2](#_Toc110486480)

[Table S2. Reducing covariate imbalance by propensity score matching 3](#_Toc110486481)

[Figure S1. Standardized % biases before and after matching 4](#_Toc110486482)

#

# Table S1. List of sites and responsible physicians

| **Site** | **Country** | **Responsible physician** | **No. of patients** |
| --- | --- | --- | --- |
| Ospedale Civile Ferrari | Italy | Dr. Bisignani, Giovanni | 34 |
| CHRU DE TOURS | France | Dr. Pierre, Bertrand | 33 |
| Royal Adelaide Hospital | Australia | Dr. Lau, Dennis H | 30 |
| Universitätsspital Zürich | Switzerland | Dr. Hofer, Daniel | 26 |
| Hospital Senhora da Oliveira - Guimarães | Portugal | Dr. Sanfins, Victor Manuel | 25 |
| Kerckhoff-Klinik GmbH | Germany | Dr. Hain, Andreas | 20 |
| Hospital Álvaro Cunqueiro | Spain | Dr. Cabanas, Pilar | 19 |
| Klinikum rechts der Isar der Technischen Universität München | Germany | Dr. Martens, Eimo | 19 |
| Centro Médico Teknon | Spain | Dr. Berruezo, Antonio | 18 |
| CHU de Brest | France | Pr. Mansourati, Jacques | 17 |
| Hôpital Gabriel Montpied, Clermont Ferrand | France | Pr. Eschalier, Romain | 14 |
| Hôpital Saint-André | France | Dr. Papaioannou, Georgios | 13 |
| Pauls Stradins Clinical University Hospital | Latvia | Pr. Erglis, Andrejs | 12 |
| Le Centre Hospitalier Universitaire de CAEN CHRU Caen | France | Pr. Milliez, Paul | 10 |
| Universitätsklinikum Gießen und Marburg GmbH, Standort Marburg | Germany | Dr. Lüsebrink, Ulrich | 10 |
| Hôpital Haut Lévêque (CHU) | France | Pr. Ploux, Sylvain | 9 |
| Charité Campus Mitte | Germany | Dr. Tscholl, Verena | 8 |
| Krankenhaus Maria-Hilf Stadtlohn | Germany | Dr. Cuneo, Alessandro | 8 |
| Centre Hospitalier Annecy Metz-Tessy | France | Dr. Dompnier, Antoine | 7 |
| CHU Montpellier | France | Pr. Pasquie, Jean-Luc | 6 |
| Hôpitaux Universitaires de Strasbourg | France | Pr. Jesel-Morel, Laurence | 5 |
| Herzzentrum Leipzig GmbH | Germany | Dr. Nedios, Sotirios | 5 |
| RHÖN-KLINIKUM Campus Bad Neustadt | Germany | Pr. Deneke, Thomas | 5 |
| Ernst-Moritz-Arndt-Universität Greifswald | Germany | Dr. Busch, Mathias | 5 |
| Semmelweis University | Hungary | Pr. Merkely, Béla | 4 |
| Hospital Universitario de Araba | Spain | Dr. Garcia, Enrique | 3 |
| Universitätsklinikum Erlangen | Germany | Dr. Arnold, Martin | 3 |
| CH Villefranche Sur Saone | France | Dr. Le Vavasseur, Olivier | 2 |
| Universitätsklinik an der Technischen Universität Dresden | Germany | Dr. Gaspar, Thomas Paul | 2 |
| **Total** |  |  | **372** |

# Table S2. Reducing covariate imbalance by propensity score matching

|  |  |  | |  |  |  |
| --- | --- | --- | --- | --- | --- | --- |
| Covariate |  | Obese group | Control group | %Bias (%) | Bias reduction (%) | P-value (t-test) |
| Mean age (years) |  |  |  |  |  |  |
| Unmatched |  | 66.0 | 60.7 | 33.8 |  | 0.006 |
| Matched |  | 66.0 | 64.5 | 9.7 | 71.2 | 0.429 |
| Female (%) |  |  |  |  |  |  |
| Unmatched |  | 51.0 | 41.3 | 19.3 |  | 0.095 |
| Matched |  | 51.0 | 51.0 | 0.0 | 100.0 | 1.000 |
| History of atrial fibrillation (%) | |  |  |  |  |  |
| Unmatched |  | 20.2 | 10.9 | 25.8 |  | 0.019 |
| Matched |  | 20.2 | 14.4 | 16.0 | 37.9 | 0.274 |
| Hypertension (%) | |  |  |  |  |  |
| Unmatched |  | 77.9 | 46.6 | 67.9 |  | <0.001 |
| Matched |  | 77.9 | 79.8 | -4.2 | 93.8 | 0.736 |
| Sleep apnoea (%) | |  |  |  |  |  |
| Unmatched |  | 16.3 | 4.9 | 37.7 |  | <0.001 |
| Matched |  | 16.2 | 18.3 | -6.3 | 83.2 | 0.716 |
| Diabetes (%] | |  |  |  |  |  |
| Unmatched |  | 23.1 | 11.3 | 31.6 |  | 0.004 |
| Matched |  | 23.1 | 16.3 | 18.0 | 43.0 | 0.224 |
| Parasternal device inclination (%)^1^ | |  |  |  |  |  |
| Unmatched |  | 38.5 | 32.7 | 12.0 |  | 0.296 |
| Matched |  | 38.5 | 35.6 | 6.0 | 49.9 | 0.668 |
| Overall |  |  |  |  |  |  |
| Unmatched |  |  |  | 32,6 |  |  |
| Matched |  |  |  | 8.6 | 73.6 |  |

^1^As opposed to “along heart axis”.

# Figure S1. Standardized % biases before and after matching

AF: atrial fibrillation.
